# Supplementary material for: Heat adaptation of phage T7 under an extended genetic code
Source: Virus Evol. 2021 Dec 1;7(2):veab100. doi: 10.1093/ve/veab100 (PMC8923235; doi:10.1093/ve/veab100)
Supplement: veab100_Supp [file veab100_supp.zip › Sequencing_Analysis.zip!/Sequencing_Analysis/A1/evidence/SNP_6.html]

BRESEQ :: Evidence


| Predicted mutation | | | | | | |
| --- | --- | --- | --- | --- | --- | --- |
| evidence | seq id | position | mutation | annotation | gene | description |
| RA | NC\_001604.1 | 21,736 | G→T | intergenic (–/–) | – / – | –/– |

| Read alignment evidence... | | | | | | | | | | | |
| --- | --- | --- | --- | --- | --- | --- | --- | --- | --- | --- | --- |
|  | seq id | position | | ref | new | freq | score (cons/poly) | reads | annotation | genes | product |
| \* | NC\_001604.1 | 21,736 | 0 | G | T | 99.0% | 6748.2 / NA | 3038 | intergenic (–/–) | *–/–* | –/– |
| Reads supporting (aligned to +/- strand):  **ref** base G (13/17);  **new** base T (1630/1370);  **total** (1649/1389) | | | | | | | | | | | |
| Fisher's exact test for biased strand distribution *p*-value = 2.70e-01 | | | | | | | | | | | |
| Kolmogorov-Smirnov test that lower quality scores support variant *p*-value = 1.00e+00 | | | | | | | | | | | |

Only 100 of 3063 total aligned reads displayed.  

`CTGGTACGAGTGCTCTTGAAGCAACTACAAGCCACGCAACAGATTCCTGAGTTACCTAAGGAAGCCGTAGAGCCAACCATTAGTACAGGTCTGGAAGCAATTGGTCGAGGACAAGACCTTGATAAGCTGGAGCGGTGTGTCACTGCGTGGGCTGCACTGGCACCTATGCGGGACGACCCTGATATTAACCTTGCGATGATTAAGTTACGTATTGCCAACGCTATCGGTATTGACACTTCTGGTATTCTACTCACCGAAGAACAGAAGCAACAGAAGATGGCCCAACAGTCTATGCAAATGGGTATGGATAATGGTGCTGCTGCGCTGGCTCAAGGTATGGCTGCACAAGCTACAGCTTCACCTGAGGCTATGGCTGCTGCCGCTGATTCCGTAGGTTTACAGCCGGGAATTTAATACGACTCACTATAGGGAGACCTCATCTTTGAAATGAGCGATGACAAGAGGTTGGAGTCCTCGGTCTTCCTGTAGTTCAACTTTAAGGAGACAATAATAATGGCTGAATCTAATGCAGACGTATATGCATCTTTTGGCGTGAACTCCGCTGTGATGTCTGGTGGTTCCGTTGAGGAACATGAGCAG  >  NC_001604.1/21437‑22036`  

                                                                                                                                                                                                                                                                                                           `|`                                                                                                                                                                                                                                                                                                              
        `caggCTCTTGAAGCAACTACAAGCCACGCAACAGATTCCTGAGTTACCTAAGGAAGCCGTAGAGCCAACCATTAGTACAGGTCTGGAAGCAATTGGTCGAGGACAAGACCTTGATAAGCTGGAGCGGTGTGTCACTGCGTGGGCTGCACTGGCACCTATGCGGGACGACCCTGATATTAACCTTGCGATGATTAAGTTACGTATTGCCAACGCTATCGGTATTGACACTTCTGGTATTCTACTCACCGAAGAACAGAAGCAACAGAAGATGGCCCAACAGTCTATGCAAATTGGTATGGAt                                                                                                                                                                                                                                                                                                     <  1:63924/298‑1 (MQ=255)`  
        `aGTGCTCTTGAAGCAACTACAAGCCACGCAACAGATTCCTGAGTTACCTAAGGAAGCCGTAGAGCCAACCATTAGTACAGGTCTGGAAGCAATTGGTCGAGGACAAGACCTTGATAAGCTGGAGCGGTGTGTCACTGCGTGGGCTGCACTGGCACCTATGCGGGACGACCCTGATATTAACCTTGCGATGATTAAGTTACGTATTGCCAACGCTATCGGTATTGACACTTCTGGTATTCTACTCACCGAAGAACAGAAGCAACAGAAGATGGCCCAACAGTCTATGCAAATTGGTATGGAt                                                                                                                                                                                                                                                                                                     <  1:30787/301‑1 (MQ=255)`  
              `ctTGAAGCAACTACAAGCCACGCAACAGATTCCTGAGTTACCTAAGGAAGCCGTAGAGCCAACCATTAGTACAGGTCTGGAAGCAATTGGTCGAGGACAAGACCTTGATAAGCTGGAGCGGTGTGTCACTGCGTGGGCTGCACTGGCACCTATGCGGGACGACCCTGATATTAACCTTGCGATGATTAAGTTACGTATTGCCAACGCTATCGGTATTGACACTTCTGGTATTCTACTCACCGAAGAACAGAAGCAACAGAAGATGGCCCAACAGTCTATGCAAATTGGTATGGATAATGGt                                                                                                                                                                                                                                                                                               <  1:479153/301‑1 (MQ=255)`  
              `ctTGAAGCAACTACAAGCCACGCAACAGATTCCTGAGTTACCTAAGGAAGCCGTAGAGCCAACCATTAGTACAGGTCTGGAAGCAATTGGTAGAGGACAAGACCTTGATAAGCTGGAGCGGTGTGTCACTGCGTGGGCTGCACTGGCACCTATGCGGGACGACCCTGATATTAACCTTGCGATGATTAAGTTACGTATTGCCAACGCTATCGGTATTGACACTTCTGGTATTCTACTCACCGAAGAACAGAAGCAACAGAAGATGGCCCAACAGTCTATGCAAATTGGTATGGATAATGGt                                                                                                                                                                                                                                                                                               <  1:27415/301‑1 (MQ=255)`  
                 `gAAGCAACTACAAGCCACGCAACAGATTCCTGAGTTACCTAAGGAAGCCGTAGAGCCAACCATTAGTACAGGTCTGGAAGCAATTGGTCGAGGACAAGACCTTGATAAGCTGGAGCGGTGTGTCACTGCGTGGGCTGCACTGGCACCTATGCGGGACGACCCTGATATTAACCTTGCGATGATTAAGTTACGTATTGCCAACGCTATCGGTATTGACACTTCTGGTATTCTACTCACCGAAGAACAGAAGCAACAGAAGATGGCCCAACAGTCTATGCAAATTGGTATGGATAATGGtgct                                                                                                                                                                                                                                                                                            >  1:408839/1‑301 (MQ=255)`  
                   `aGCAACTACAAGCCACGCAACAGATTCCTGAGTTACCTAAGGAAGCCGTAGAGCCAACCATTAGTACAGTTCTGGAAGCAATTGGTCGAGGACAAGACCTTGATAAGCTGTAGCGGTGTGTCACTGCGTGGGCTGCACTGGCACCTATGCGGGACGACCCTGATATTAACCTTGCGATGATTAAGTTACGTATTGCCAACGCTATCGGTATTGACACTTCTGGTATTCTACTCACCGAAGAACAGAAGCAACAGAAGATGGCCCAACAGTCTATGCAAATTGGTGTGGATAATGGtgctgc                                                                                                                                                                                                                                                                                          <  1:324988/301‑1 (MQ=255)`  
                       `aCTACAAGCCACGCAACTGATTCCTGAGTTACCTAAGGAAGCCGTAGAGCCAACCATTAGTACAGGTCTGGAAGCAATTGGTCGAGGACAAGACCTTGATAAGCTGGAGCGGTGTGTCACTGCGTGGGCTGCACTGGCACCTATGCGGGACGACCCTGATATTAACCTTGCGATGATTAAGTTACGTATTGCCAACGCTATCGGTATTGACACTTCTGGTATTCTACTCACCGAAGAACAGAAGCAACAGAAGATGGCCCAACAGTCTATGCAAATTGGtttctgtctcttatacacatct                                                                                                                                                                                                                                                                                      >  1:367440/1‑280 (MQ=255)`  
                          `aCAAGCCACGCAACAGATTCCTGAGTTACCTAAGGAAGCCGTAGAGCCAACCATTAGTACAGGTCTGGAAGCAATTGGTCGAGGACAAGACCTTGATAAGCTGGAGCGGTGTGTCACTGCGTGGGCTGCACTGGCACCTATGCGGGACGACCCTGATATTAACCTTGCGATGATTAAGTTACGTATTGCCAACGATATCGGTATTGACACTTCTGGTATTCTACTCACCGAAGAACAGAAGCAACAGAAGATGGCCCAACAGTCTATGCAAATTGGTATGGATAATGGTGCTGCTGCGCTg                                                                                                                                                                                                                                                                                   <  1:383045/301‑1 (MQ=255)`  
                               `ccACGCAACAGATTCCTGAGTTACCTAAGGACGCCGTAGAGCCAACCATTAGTACAGATCTGGAAGCAATTGGTCGAGGACAAGACCTTGATAAGCTGGAGCGGTGTGTCACTTCGTGGGCTGCACTGGCACCTATGCATGGCGAACCTGATCTTAACCTTGCGCTGATTAAGTTACGTATTGACAACGCTCCCGGTATTGACACTTCTGGTATTCTACTCACCGAAGAACAGAAGCAACAGAAGATGGCCAAACAGTCTCTGCAAATGGGTATGGAAAATGGTGCTGCTGCGCTAGCTCa                                                                                                                                                                                                                                                                              >  1:112064/1‑301 (MQ=255)`  
                                  `cGCAACAGATTCATGAGTTACATAAGGAAGCCGTAGAGCAAACCATTAGTACAGGTCTGGAAGCAATTGGTCGAGGACAAGACCGTGATAAGCTGGAGCGGTGTGTCACTGCGTGGGCTGCACTGGCACCTATGCGGGACGACCCTGATATTAACCTTGCGATGATTAAGTTACGTATTGCCAACGCTATCGGTATTGACACTTCTGGTATTCTACTCACCGAAGAACAGAAGCAACAGAAGATGGCCCAACAGTCTATGCAAATTGGTATGGATAATGGTGCTGCTGCGCTGGCTCAAgg                                                                                                                                                                                                                                                                           <  1:219150/301‑1 (MQ=255)`  
                                   `agacaggATTCCTGAGTTACCTAAGGAAGCCGTAGAGCCAACCATTAGTACAGGTCTGGAAGCAATTGGTCGAGGACAAGACCTTGATAAGCTGGAGCGGTGTGTCACTGCGTGGGCTGCACTGGCACCTATGCGGGACGACCCTGATATTAACCTTGCGATGATTAAGTTACGTATTGCCAACGCTATCGGTATTGACACTTCTGGTATTCTACTCACCGAAGAACAGAAGCAACAGAAGATGGCCCAACAGTCTATGAAAATTGGTATGGATAATGGTGCTGCTGCGCTGGCTCAAGGt                                                                                                                                                                                                                                                                          <  1:363994/295‑1 (MQ=255)`  
                                    `cAACAGATTCCTGAGTTACCTAAGGAAGCCGTAGAGACAACCATTAGTACAGGTCTGGAAGCAATTGGTCGAGGACAAGACCTTGATAAGCTGGAGCGGTGTGTCACTGCGTGGGCTGCACTGGCACCTATGCGGGACGACCCTGATATTAACCTTGCGATGATTAAGTTACGTATTGCCAACGCTATCGGGATTGACACTTCTGGTATTCTACTCACCGAAGAACAGAAGCAACAGAAGATGGTCCAACAGTCTATGCAAATTGGTATGGATAATGCTGCTGCTGCGCTGGCCAAAggaa                                                                                                                                                                                                                                                                         >  1:352910/1‑299 (MQ=255)`  
                                      `aCAGATTCCTGAGTTACCTAAGGAAGCCGTAGAGCCAACCATTAGTACAGGTCTGGAAGCAATTGGTCGAGGACAAGACCTTGATAAGCTGGAGCGGTGTGTCACTGCGTGGGCTGCACTTGCACCTATGCGGGACGACCCTGATATTAACCTTGCGATGATTAAGTTACGTATTGCCAACGCTGTCGGTATTGACACTTCTAGTACTCTACTCACCGAAGAACAGAAGCAACAGAAGATGGCCCAACAGTCTATGCAAATTGGTATGGATAATGGTGCTGCTGCGCTGGCTCAAGGTATg                                                                                                                                                                                                                                                                       >  1:226937/1‑301 (MQ=255)`  
                                        `gtgggctcggagatgtgtataagagacagtagCCAACCATTAGTACAGGTCTGGAAGCAATTGGTCGAGGACAAGACCTTGATAAGCTGGAGCGGTGTGTCACTGCGTGGGCTGCACTGGCACCTATGCGGGACGACCCTGATATTAACCTTGCGATGATTAAGTTACGTATTGCCAACGCTATAGGTATTGACACTTCTGGTATTCTACTCACCGAAGAACAGAAGCAACAGAAGATGGCCCAACAGTCTATGCAAATTGGTATGGATAATGGTGCTGCTGCGCTGGCTCAAGGTATGGc                                                                                                                                                                                                                                                                     <  1:339837/271‑1 (MQ=255)`  
                                        `aaagtctggAGTTAGCGAAAGAAGACTTAGAGGCAACAATTAGTACAGGGCTGTAATCAATTGGTCGATGACAAGACCGAGATAAGCTGGAGCGGTGTGGCACTGCGTGGTCTGTACTGGCACCTATGCGGGACGACCCTGATATTAACCTTGCGATGATTAAGTTATGTAATGCCAACGCTATCGGTATTGACACTTCTGGTATTCTACTCACCGAAGAACAGAAGCAACAGAAGATGGCCCAACAGTCTATGCAAATTGGTATGGATAATGGTGCTGCTGCGCTGGCTCAAGGTATGGc                                                                                                                                                                                                                                                                     <  1:68599/293‑1 (MQ=255)`  
                                                     `aCCTAAGGAAGCCGTAGAGCCAACCATTAGTACAGGTCTGGAAGCAATTGGTCGAGGACAAGACCTTGATAAGCTGGAGCGGTGTGTCACTGCGTGGGCTGCACTGGCACCTATGCGGGACGACCCTGATATTAACCTTGCGATGATTAAGTTACGTCTTGCCAACGCTATCGGTATTGACACTTCTGGTATTCTACTCACCGAAGACCAGAAGCAACAGAAGATGGCCCAACAGTCTATGCAAATTGGTATGGATAATGGTGCTGCTGCGCTGGCTCAAGGTATGGcctgtctcttataa                                                                                                                                                                                                                                                        >  1:296007/1‑288 (MQ=255)`  
                                                                   `tAGAGCCAACCATTAGTACAGGTCTGGAAGCAATTGGGCGAGGACAAGACCTTGATAAGCTGGAGCGGTGTGTCACTGCGTGGGCTGCACTGGCACCTATGCGGGACGACCCTGATATTAACCTTGCGATGATTAAGTTACGTATTGCCAACGCTATCGGTATTGACACTTCTGGTATTCTACTCACCGAAGAACAGAAGCAACAGAAGATGGCCCAACAGTCTATGCAAATTGGTATGGATAATGGTGCTGCTGCGCTGGCTCAAGGTATGGCTGCACAAGCTACAGCTTCACCTGAGGc                                                                                                                                                                                                                                          <  1:440688/301‑1 (MQ=255)`  
                                                                     `gagCCAACCATTAGTACAGGTCTGGAAGCAATTGGTCGAGGACAAGACCTTGATAAGCTGGAGCGGTGTGTCACTGCGTGGGCTGCACTGGCACCTATGCGGGACGACCCTGATATTAACCTTGCGATGATTAAGTTACGTATTGCCAACGCTATCGGTATTGACACTTCTGGTATTCTACTCACCGAAGAACAGAAGCAACAGAAGATGGCCCAACAGTCTATGCAAATTGGTATGGATAATGGTGCTGCTGCGCTGGCTCAAGGTATGGCTGCCCAAGCTACAGCTTCACCTGAGGCtt                                                                                                                                                                                                                                        >  1:466482/1‑300 (MQ=255)`  
                                                                        `ccAAACATTAGTACAGGTCTGGAAGCAATTGGTCGAGGACAAGACCTTGATAAGCTGGAGCGGTGTGTCACTGCGTGGGCTGCACTGGCACCTATGCGGGACGACCCTGATATTAACCTTGCGATGATTAAGTTACGTAGTGCCAACGCTAGCGGTATTGACACTTCTGGTATTCTACTCACCGAAGAACAGAAGCAACAGAAGATGGCCCAACAGTATATGCAAATTGGTATGGATAATGATGCTGCTGCGCTGGCTCAAGGTATGGCTGCACAAGCTACAGATTCACCTGAGGCTATgg                                                                                                                                                                                                                                     <  1:1849/301‑1 (MQ=255)`  
                                                                            `ccATTAGTACAGGTCTGGAAGCAATTGGTCGAGGACAAGACCTTGATAAGCTGGAGCGGTGTGTCACTGCGTGGGCTGCACTGGGACCTATGCGGGACGACCCTGATATTAACCTTGCGATGATTAAGTTACGTATTGCCAACGCTATCGGTATTGACACTTCTGGTATTCTACTCACCGAAGAACAGAAGCAACAGAAGATGGCCCAACAGTCTATGCAAATTGGTATGGATAATGGTGCTGCTGCGCTGGCTCAAGGTATGGCTGCCCAAGcagccataccttgagccagcgcagcagc                                                                                                                                                                                                                                 >  1:404134/1‑274 (MQ=255)`  
                                                                            `ccATTAGTACAGGTCTGGAAGCAATTGGTCGAGGACAAGACCTTGATAAGCTGGAGCGGTGTGTCACTGCGTGGGCTGCACTGGCACCTATGCGGGACGACCCTGATATTAACATTGCGATGATTAAGTTACGTATTGCCAACGCTATCGGTATTGACACTTCAGGTATTCTACTCACCGAAGAACAGAAGCAACAGAAGATGGCCCAACAGTCTATGCAAATTGGTATGGATAATGGTGCTGCTGCGCTGGCTCAAGGTATGGCTGCACAAGCTACAGCTTCACCTGAGGCTCTGtctct                                                                                                                                                                                                                                 >  1:88283/1‑299 (MQ=255)`  
                                                                             `cATTAGTACAGGTCTGGAAGCAATTGGTCGAGGACAAGACCTTGATAAGCTGGAGCGGTGTGTCACTGCGTGGGCTGCACTGGCACCTATGCGGGACGACCCTGATATTAACCTTGCGATGATTAAGTTACGTATTGCCAACGCTATCGGTATTGACACTTCTGGTATTCTACTCACCGAAGAACAGAAGCAACAGAAGATGGCCCAACAGTCTATGCAAATTGGTATGGATAATGGTGCTGCTGCGCTGGCTCAAGGTATGGCTGCACACGCTACAGCTTCACCTGAGGCTATGgctgct                                                                                                                                                                                                                                >  1:54265/1‑301 (MQ=255)`  
                                                                                  `gTACAGGTCTGGAAGCAATTGGTCGAGGACAAGACCTTGATAAGCTGGAGCGGTGTGTCACTGCGTGGGCTGCACTGGCACCTATTCGGGACGACCCTGATATTAACCTTGCGATGATTAAGTTACGTATTGCCAACGCTATCGGTATTGACACTTCTGGTATTCTACTCACCGAAGAACAGAAGCAACAGAAGATGGCCCAACAGTC‑‑TGCAAATTGGTATGGATAATGGTGCTGCTGCGCTGGCTCAAGGTATGGCTGCACAAGCTACAGCTTCACCTGAGGCTactgtctcttatacac                                                                                                                                                                                                                         >  1:107912/1‑286 (MQ=255)`  
                                                                                                     `ggataagagACAGGACCTTGATAAGCTGGAGCGGTGTGTCACTGCGTGGGCTGCACTGGCACATATGCGGGACGACCCTGATATTAACCTTGCGATGATTAAGTTACGTATTGCCAACGCTATCGGTATTGACACTTCTGGTATTCTACTCACCGAAGAACAGAAGAAACAGAAGATGGCCCAACAGTCTATGCAAATTGGTATGGATAATGGTGCTGCTGCGCTGGCTCAAGGTATGGCTGCACAAGCTACAGCTTCACCTGAGGCTATGGCTGCTGCCGCTGATTCCGTAGGTTTACAg                                                                                                                                                                                                        <  1:206664/293‑1 (MQ=255)`  
                                                                                                       `gTCGAGGACAAGACCTTGATAAGCTGGAGCGGTGTGTCACTGCGTGGGCTGCACTGGCACCTATGCGGGACGACCCTGATATTAACCTTGCGATGATTAAGTTACGTATTGCCAACGCTATCGGTATTGACACTTCTGGTATTCTACTCACCGAAGAACAGAAGCAACAGAAGATGGCCCAACAGTCTATGCAAATTGGTATGGATAATGGTGCTGCTGCGCTGGCTCAAGGTATGGCTTCACAAGCTACAGCTTCACCTGAGGCTATGGCTGCTGCCGCTGCTTCCGTAGGTTTACAGcc                                                                                                                                                                                                      >  1:255278/1‑301 (MQ=255)`  
                                                                                                            `ggACAAGACCTTGATAAGCTGGAGCGGTGTGTCACTGCGTGGGCTGCACTGGCACCTATGCGGGACGACCCTGATATTAACCTTGCGATGATTAAGTTACGTATTGCCAACGCTATCGGTATTTACACTTCTGGTATTCTACTCACCGAAGAACAGAAGCAACAGAAGATGGCCCAACAGTCTATGCAAATTGGTATGGATAATTGTGCTGCTGCGCTGGCTCAAGGTATGGCTGCACAAGCTACAGCTTCACCTGAGGCTATGGCTGCTGCCGCTGATTCCGTAGGTTTACATCCGGGaa                                                                                                                                                                                                 >  1:461664/1‑301 (MQ=255)`  
                                                                                                             `gACAAGACCTTGAGAAGCTGGAGCGGTGTGTCACTGCGTGGGCTGCACGGGCACCTATGCGGGACGACCCTGATATTAACCTTGCGATGATTAAGTTACGTATTGCCAACGCTATCGGTATTGACACTTCTGGTATTCTACTCACCGAAGAACAGAAGCAACAGAAGATGGCCCAACAGTCTATGCAAATTGGTATGGATAATGGTGCTTCTGCGCTGGCTCAAGGTATGGCTGCACAAGCTACAGCTTCACCTGAGGCTATGGCTGCTGCCGCTGATTCCGTAGGTTTACAGCCGGAAAt                                                                                                                                                                                                <  1:125946/301‑1 (MQ=255)`  
                                                                                                                       `ttataagaaacaGCGGTGTGTCACTGCGTGGGCTGCACTGGCACCTATGCGGGACGACCCTGATATTAACCTTGCGATGATTAAGTTACGTATTGCCAACGCTATCGGTATTGACACTTCTGGTATTCTACTCACCGAAGAACAGAAGCAACAGAAGATGGCCCAACAGTCTATGCAAATTGGTATGGATAATGGTGCTGCTGCGCTGGCTCAAGGTATGTCTGCACAAGCTACAGCTTCACCTGAGGCTATGGCTGCTGCCGCTGATTCCGTAGGTTTACAGCCGGGAATTTAATACGAc                                                                                                                                                                                      <  1:329209/290‑1 (MQ=255)`  
                                                                                                                           `aaGCTGGAGCGGTGTGTAACTGCGTGGGCTGCACTGGCACCTATGCGGGACGACCCTGATATTAACCTTGCGATGATTAAGTTACGTATTGCCAACGCTATCGGTATTGACACTTCTGGTATTCTACTCACCGAAGAACAGAAGCAACAGAAGATGGCCCAACAGTCTATGCAAATTGGTATGGATAATGGTGCTGCTGCGCTGGCTCAAGGTATGGCTGCACAAGCTACAGCTTCACCTGAGGCTATGGCTGCTGCCGCTGATTCCGTAGGTTTACAGCCGGGAATTTAATACGACTAAc                                                                                                                                                                                  >  1:92503/1‑301 (MQ=255)`  
                                                                                                                             `gCTGGAGCGGTGTGTCACTGCGTGGGCTGCACTGGCACCTATGCGGGACGACCCTGATATTAACCTTGCGATGATTAAGTTACGTATTGCCAACGCTATCGGTATTGACACTTCTGGTATTCTACTCACCGAAGAACCGAAGCAACAGAAGATGGACCAACAGTCTATGCAAATTGGTATGGATACTGGTGCTGCTGCGCTGGCTCAAGGTATGGCTGCACAAGCTACAGCTTCACCTGAGGCTATGGCTTCTGCCGCTGATTCCGTAGGTTTACAGCCGGGAATTTAATACGACTCACta                                                                                                                                                                                >  1:234463/1‑301 (MQ=255)`  
                                                                                                                             `gCTGGAGCGGTGTGTCACTGCGTGGGCTGCACTGGCACCTATGCGGGACGACCCTGATATTAACCTTGCGATGATTAAGTTACGTATTGCCAACGCTATCGGTATTGACACTTCTGGTATTCTACTCACCGAAGAACAGAAGCAACAGAAGATGGCCCAACAGTCTATGCAAATTGGTATGGATAATGGTGCTGCTGCGCTGGCTCAAGGTATGGCTGCACAAGCTACAGCTTCACCTGAGGCTATGGCTTCTGCCGCTGATTCCGTAGGTTTACAGCCGGGAATTTAATACGACTCACta                                                                                                                                                                                >  1:430934/1‑301 (MQ=255)`  
                                                                                                                              `cTGGAGCGGTGTGTCACTGCGTGGGCTGCACTGGCACCTATGCGGGACGACCCTGATATTAACCTTGCGATGATTAAGTTACGTATTGCCAACGCTATCGGTATTGACACTTCTGGTATTCTACTCACCGAAGAACAGAAGCAACAGAAGATGGCCCAACAGTTTATGCAAATTGGTATGGATAATGGTGCTGCTGCGTTGGCTCAAGGTATGGCTGCACAAGCTACAGCTTCACCTGAGGCTATGGCTGCTGCCGCTGATCCCGTAGGTTTACAGCCGGGAATTTAAGACGACTCACtat                                                                                                                                                                               <  1:237601/301‑1 (MQ=255)`  
                                                                                                                              `cTGGAGCGGTGTGTCACTGCGTGGGCTGCACTGGCACCTATGCGGGACGACCCTGATATTAACCTTGCGATGATTAAGTTACGTATTGCCAACGCTATCGGTATTGACACTTCTGGTATTCTACTCACCGAAGAACAGAAGCAACAGAAGATGGCCAAACAGTCTATGCAAATTGGTATGGATAATGGTGCTGCTGCGCTGGCTCAAGGTATGGCTGCACAAGCTACAGCTTCACCTGAGGCTATGGCTGCTGCCACTGATTCCGTAGGTTTACAGCCGGGAATTTAATACGACTCACtat                                                                                                                                                                               <  1:264552/301‑1 (MQ=255)`  
                                                                                                                                 `gtgCGGTGTGTCACTGCGTGTGCTGCACTGGCACCTATGCGGGACGACCCTGATATTAACCTTGCTATGATTAAGTTACGGATTGCCAACGCTATCGGTATTGACACTTCTGGTATTCTACTCACCGAAGAACAGAAGCAACAGAAGATGGCCCAACAGTCTATGCAAATTGGTATGGATAATGGTGCTGCTGCGCTGGCTCAAGGTATGGCTGCACAAGCTACAGCTTCACCTGAGGCTATGGCTGCTGCCGCTGATTCCGTAGGTTTACAGCCGGGAATTTAATACGACTCACTATAgg                                                                                                                                                                            <  1:278134/299‑1 (MQ=255)`  
                                                                                                                                  `ggCGGTGTGTCACTGCGTGGGCTGCACTGGCACCTATGCGGGACGACCCTGATATTAACCTTGCGATGATTAAGTTACGTATTGCCAACGCTATCGGTATTGACACTTCTGGTATTCTACTCACCGAAGAACAGAAGCAACAGAAGATGGCCCAACAGTCTATGAAAATTGGTATGGATAATGGTGCTGCTGCGCTGGCTCAAGGTATGGCTGCACAAGCTACAGCTTCACCTGAGGCTATGGCTGCTGCCGCTGATTCCGTAGGTTTACAGCCGGGAATTTAATACGACTCACTATAggg                                                                                                                                                                           <  1:230313/300‑1 (MQ=255)`  
                                                                                                                                  `aGCGGTGTGGCACTGCGTGGGCTGCACTGGTACCTATACGGGACTACCCTGATATTAACCTTGCGTTGATTAAGTTACGTCTTGCCAACGCTGTCTGTATTGACACTTAGTGTATTCTACTCACCGAAGATCAGAAGCACCTGAAGATGGCCCAACAGTCTATGCAAATTGGTATGGATAATGGTGCTGCTGCGCTGGCTCAATGTATGGCTGCACAAGCTACAGCTTCACCTGAATCTATGGCTGCTGCCGCTGATTCCGTATGTTTACAGCCGGGAATTTAATACGACTCACTATAggg                                                                                                                                                                           <  1:14511/301‑1 (MQ=255)`  
                                                                                                                                   `gCGGTGTGTCACTGAGTGGGCTGCACTGGCACCTATGCGGGACGACCCTGATATTAACCTTGCGATGATTAAGTTACGTATTGCCAACGATATCGGTATTGACACTTCTGGTATTCTACTCACCGAAGAACAGAAGCAACAGAAGATGGCCCAACAGTCTATGCAAATTGGTATGGATAATGGTGCTGCTGCGCTGGCTCAAGGTATGGCTGCACAAGCTACAGCTTCACCTGAGGCTATGGCTGCTGCCGCTGATTCCGTAGGTTTACAGCCGGGAATTTAATACGACTCACTATAGGga                                                                                                                                                                          <  1:180203/301‑1 (MQ=255)`  
                                                                                                                                    `gggTGGGTCACTGCGTGGGCTGCACTGGCACCTATGCGGGACGACCCGGATATTAAACTTGCGATGATTAAGTTACGTATTGCAAACGCTATAGGTATTGACACGTCTGGTATTCTACTCACCGAAGAAAAGAAGCAACAGAAGATGGCCAAACAGTCTATGCAAAGTGGTATGGATAATGGTGCTGCTGCGCTGGCTCAAGGTAGGGCTGCACAAGCTACAGCTTCACCTGAGGCTATGGCTGCTGCCGCGGATTCCGTAGGTTTACAGCCGGGAATTTAATACGACTCACTATAGGgag                                                                                                                                                                         <  1:379403/300‑1 (MQ=255)`  
                                                                                                                                    `aggTGTGTCACTGCGTGGGCTGCACTGGAAACTATGCGGGACGACCCTGATATTAACCTTGCGATGATTAAGTTACGTATTGCCAACGCTATCGGTATTGACACTTCTGGTATTCTACTCACCGAAGAACAGAAGCAACAGAAGATGGCCCAACAGTCTATGCAAATTGGTATGGATAATGGTGCTGCTGCGCTGGCTCAAGGTATGGCTGCACAAGCTACAGCTTCACCTGAGGCTATGGCTGCTGCCGCTGATTCCGTAGGTTTACAGCCGGGAATTTAATACGACTCACTATAGGgag                                                                                                                                                                         <  1:358632/300‑1 (MQ=255)`  
                                                                                                                                     `ggTGTGTCACTGCGTGGGCTGCACTGGCACCTATGCGGGACGACCCTGATATTAACCTTGCGATGATTAAGTTACGTATTGCCAACGCTATCGGTATTGACACTTCTGGTATTCTACTCACCGAAGAACAGAAGCAACAGAAGATGGCCCACCATTCTATGCAAATTGGTATGGATAATGGTGCTGCTGCGCTGGCTCAATGTATGGCTGCACAACCTACAGCTTCACCTGAGGCTATGGCTGCTGCCGCTGATTCCGTAGGTTTACAGCCGGGCATTTAATACGACTCACTATAggtact                                                                                                                                                                        >  1:495788/1‑297 (MQ=255)`  
                                                                                                                                     `ggTGTGTCACTGCGTGGGCTGCACTGGCACCTATGCGGGACGACCCTGATATTAACCTTGCGATGATTAAGTTACGTATTGCCAACGCTATCGGTATTGACACTTCTGGTATTCTACTCACCGAAGAACAGAAGCAACAGAAGATGGCCCAACAGTCTATGCAAATTGGTCTGGATAATGGTGCTGCTGCGCTGGCTCAAGGTATGGCTGCACAAGCTACAGCTTCACCTGAGGCTATGGCTGCTGCCGCTGATTCCGTAGGTTTACAGCCGGGAATTTAATCCGACTCACTATAGGgaga                                                                                                                                                                        >  1:284246/1‑301 (MQ=255)`  
                                                                                                                                      `gtgtgtCACTGCGTGTGCTGCACTGGCACCTATGCGGGACGACCCTGATATTACCCTTGCGATGATTCAGTTACGTCTTGCCAACGCTATCGGTCTTGACACTTCTGGTATTCTACTCACCGAAGAACAGAAGCATCAGAAGATGGCCCATCAGTCTATGCAACTTGCTATGGACAATGGTGCTGCTTCGCTGGCTCAATGCCTGGCTGCACCAGCCACAGTTTCACCTGAGGCTATGGCTGCTGCCTCTGATTCCGTTGATTTACAGCCCTGACTTCACTACGACTCACTATATGGAGAc                                                                                                                                                                       >  1:302023/1‑301 (MQ=255)`  
                                                                                                                                       `ggtgtCACTGCGTGGGCTGAACTGGCCCCTAGGCGGGACGACCCTGATATTAACCTTGCGATGATTAAGTTACGTATTGCCAACGCTATCGGTATTGACACTTCTGGTATTCTACTCACCGAAGAACAGAAGCAACAGAAGATGGCCCAACAGTCTATGCAAATTGGTATGGATAATGGTGCTGCTGCGCTGGCTCAAGGTATTGCTGCACAAGCTACAGCTTCACCTGAGGCTATGGCTGCTGCCGCTGATTCCGTAGGTTTACAGCCGGGAATTTAATACGACTCACTATAGGGAGAcc                                                                                                                                                                      <  1:82654/300‑1 (MQ=255)`  
                                                                                                                                        `gtgtCACTGCGTGGGCTGCACTGGCACCTATGCGGGACGACCCTGATATTAACCTTTCGATGATTAAGTTACGTATTGCCAACGCTATCGGTATTGACACTTCTGGTATTCTACTCACCGAAGAACAGAAGCAACAGAAGATGGCCCAACAGTCTATGCAAATTGGTATGGATAATGGTGCTGCTGCGCTGGCTCAAGGTATGGCTGCACAAGCTACAGCTTCACCTGAGGCTATGGCTGCTGCCGCTGATTCCGTAGGTTTACAGCCGGTAATTTAATACGACTCACtatctgtctcttt                                                                                                                                                                     >  1:388263/1‑291 (MQ=255)`  
                                                                                                                                          `gtCACTGCGTGGGCTGCACTGGCACCTATGCGGGACGACCCTGATATTAACCTTGCGATGATTAAGTTACGTATTGCCAACGCTATCGGTATTGACACTTCTGGTCTTCTACTCACCGAAGAACAGAAGCACCAGAAGATGGCCCAACAGTCTATGCAACTTGGTATGGATAATGGTGCTGCTGCGCTGGCTCAAGGTATGGCTGCACACGCTACAGCTTCACCTGAGGCTATGGCTGCTGCCGCTGATTCCGTAGGTTTACAGCCGGGAATTTAATACGACTCACTATAGGGAGACCGCa                                                                                                                                                                   >  1:211634/1‑301 (MQ=255)`  
                                                                                                                                            `cACTGCGTGGGCTGCACTGGCACCTATGCGGGACGACCCTGATATTAACCTTGCGATGATTAAGTTACGTATTGCCAACGCTATCGGTATTGACACTTCTGGTATTCTACTCACCGAAGAACAGAAGCAACAGAAGATGGCCCAACAGTCTATGCAAATTGGTATGGATAATGGTGCTGCTGCGCTGGCTCAAGGTATGGCTGCACAAGCTACAGCTTCACCTGAGGCTATGGCTGCTGCCGCTGATTCCGTAGGTTTACAGCCGGGAATTTAATACGACTCACTATAggctgtctcttat                                                                                                                                                                 >  1:140780/1‑290 (MQ=255)`  
                                                                                                                                                    `gggCTGCACTGGCACCTATGCGGGACGACCCTGATATTAACCTTGCGATGATTAAGTTACGTATTGCCAACGCTATCGGTATTGACACTTCTGGTATTCTACTCAACGAAGAACAGAAGCAACAGAAGATGGCCCAACAGTCTATGCAAATTGGTATGGATAATGGTGCTGCTGCGCTGGCTCAAGGTATGGCTGCACAAGCTACAGCTTCACCTGAGGCTATGGCTGCTGCCGCTGATTCCGTAGGTTTACAGCCGGGAATTTAATACGACTCACTATAGGGAGACCTCATTTTTGAAAt                                                                                                                                                         >  1:100258/1‑301 (MQ=255)`  
                                                                                                                                                     `ggCTGCACTGGCACCTAGGCGGGAATACCCGTATATTAACCTTGCGATGATTAAGTTACGGATTGCCAACGCTATCGGTATTGACATTTATGGTATTCTAATCACCGAAGAACAGAAGCAACAGAAGATGACCCAACAGTCTATGCAAATTGGTATGGCTAATGGTGCTGCTGCGCTGGCTCAAGGTATGGCTGCACAAGCTACAGCTTCACCTGAGGCTATTGCTGCTGCCGCTGATTCCGTAGGTTTACAGCCGGGAATTTAATACGACTCACTATAGGGAGACCTCATCTTTGAAATg                                                                                                                                                        <  1:249328/301‑1 (MQ=255)`  
                                                                                                                                                                          `ggACGACCCTGATATTAACCTTGCGATGATTAAGTTACGTATTGCCAACGCTATCGGTATTGACACTTCTGGTATTCTACTCACCGAAGAACAGAAGCAACAGAAGATGGCCCAACAGTATATGCAAATTGGTATGGATAATGGTGCTGCTGCGCTGGCTCAAGGTATGGCTGCACAAGCTACAGCTTCACCTGAGGCTATGGCTGCTGCCGCTGATTCCGTAGGTTTACAGCCGGGAATTTAATACGACTCACTATAGGGAGACCTCATCTTTGaaactgtctcttatacacatctccga                                                                                                                                   >  1:189531/1‑278 (MQ=255)`  
                                                                                                                                                                               `acCCTGATATTAACCTTGCGATGATTAAGTTACGTATTGCCAACGCTATCGGTATTGACACTTCTGGTATTCTACTCACCGAAGAACAGAAGCAACAGAAGATGGCCCAACAGTCTATGCAAATTGGTATGGATAATGGTGCTGCTGCGCTGGCTCAAGGTATGGCTGCACAAGCTACAGCTTCACCTGAGGCTATGGCTGCTGCCGCTGATTCCGTAGGTTTACAGCCGGGAATTTAATACGACTCACTATAGGGAGACCTCATCTTTGAAATGAGCGATGACAAGAGGTTggctgtctt                                                                                                                              >  1:164868/1‑294 (MQ=255)`  
                                                                                                                                                                                `cccTGATATTAACCTTGCGATGATTAAGTTACGTATTGCCAACGCTATCGGTATTGACACTTCTGGTATTCTACTCACCGAAGAACAGAAGCAACAGAAGATGGCCCAACAGTCTATGCAAATTGGTATGGATAATGGTGATGCTGCGCTGGCACAAGGTATGGCTGCACACGCTACAGCTTCAACTGAGGCTATGGCTGCTGCCGCTGATTCAGTAGGATTACAGCCGGGAATTTACTCCGAATAACTATAGGGAGAACTCATCTTTGAAATGAGCGATGAAAAGAGGTTGTAGTACTCg                                                                                                                             >  1:436512/1‑301 (MQ=255)`  
                                                                                                                                                                                `cccTGATATTAACCTTGCGATGATTAAGTTACGTATTGCCAACGCTATCGGTATTGACACTTCTGGTATTCTACTCACCGAAGAACAGAAGCAACAGAAGATGGCCCAACAGTCTATGAAAATTGGTATGGATAATGGTGCTGCTGCGCTGGCTCAAGGTATGGCTGCACAAGCTACAGCTTCACCCGAGGCTATGGCTGCTGCCGCTGATTCCGTAGGTTTAAAGCCGGGAATTTAATACGACTCACTTTCGGGAGACCTCATCTTTGAAATGAGCGATGACAAGAGGTTGGAGTCATCg                                                                                                                             >  1:507551/1‑301 (MQ=255)`  
                                                                                                                                                                                  `cTGATATTAACCTTGCGATGATTAAGTTACGTATTGCCAACGATATCGGTATTGACACTTCTGGTATTCTACTCACCGAAGAACAGAAGCAACAGAAGATGGCCCAACAGTCTATGCAAATTGGTATGGATAATGGTGCTGCTGCGCTGGCTCAAGGTATGGCTGCACAAGCTACAGCTTCACCTGAGGCTATGGCTGCTGCCGCTGATTCCGTAGGTTTACAGCCGGGAATTTAATACGACTCACTATAGGGAGACCTCATCTTTGAAATGAGCGATGACAAGAGGTTGGAGTCCTCGGt                                                                                                                           <  1:474816/301‑1 (MQ=255)`  
                                                                                                                                                                                    `gATATTAACCTTGCGATGATTAAGTTACGTATTGCCAACGCTATCGGTATTGACACTTCTGGTATTCTACTCACCGAAGAACAGAAGCAACAGAAGATGGCCCAACAGTCTATGCAAATTGGTATGGATAATGGTGCTGCTGCGCTGGCTCAAGGTATGGCTGCACAAGCTACAGCTTCACCTGAGGCTATGGCTGCTGCCGCTGATTCCGTAGGTTTACAGCCGGGAATTTAATACGACTCACTATAGGGAGACCGCATCTTTGAAATGAGCGATGTCAAGAGGTTGGAGTCCTCGGTCt                                                                                                                         >  1:348476/1‑301 (MQ=255)`  
                                                                                                                                                                                    `gATATTAACCTTGCGATGATTAAGTTACGTATTGCCAACGCTATCGGTATTGACACTTCTGGTATTCTACTCACCGAAGAACAGAAGCAACAGAAGATGGCCCAACAGTCTATGCAAATTGGTATGGATAATGGTGCTGCTGCGCTGGCTCAAGGTATGGCTGCACAAGCTACAGCTTCACCTGAGGCTATGGCTGCTGCCGCTGATTCCGTAGGTTTACAGCCGGGAATTTAATACGACTCACTATAAGAAGACCTCATCTTTGAAATGAGCGATGACAAAAGGTTGGAGTCCTCGGTCt                                                                                                                         >  1:489677/1‑301 (MQ=255)`  
                                                                                                                                                                                    `gATATTAACCTTGCGATGATTAAGTTACGTATTGCCAACGCTATCGGTATTGACACTTCTGGTATTCTACTCACCGAAGAACAGAAGCAACAGAAGATGGCCCAACAGTCTATGCAAATTGGTATGGATAATGGTGCTGCTGCGCTGGCTCAAGGTATGGCTGCACAAGCTACAGCTTCACCTGAGGCTATGGCTGCTGCCGCTGATTCCGTAGGTTTACAGCCAGGAATTTAATACTACTCACTATAGGGAGACCTCATCTTTGAAATGAGCGATGACAAGAGGTTGGAGTCCTCGGTCt                                                                                                                         >  1:483947/1‑301 (MQ=255)`  
                                                                                                                                                                                    `gATATTAACCTTGCGATGATTAAGTTACGTATTGCCAACGCTATCGGTATTGACACTTCTGGTATTCTACTCACCGAAGAACAGAAGCAACAGAAGATGGCCCAACAGTCTATGCAAATTGGTATGGATAATGGTGCTGCTGCGCTGGCCCAAGGTATGGCTGCACAAGCTACAGCTTCACCTGAGGCTATGGCTGCTGCCGCTGATTCCGTAGGTTTACAGCCGGGAATTTAATACGACTCACTATAGGCCGACCTCATCTTTGAAATGAGAGATGACAAGAGGTTGTAGTCCTCGGTCt                                                                                                                         >  1:17069/1‑301 (MQ=255)`  
                                                                                                                                                                                    `gATATTAACCTTGCGATGATTAAGTTACGTATTGCCAACGCTATCGGTATTGACACTTCTGGTATTCTACTCACCGAAGAACAGAAGCAACAGAAGATGGCCCAACAGTCTATGCAAATTGGTATGGATAATGGTGCTGCTGCGATGGCTCAAGGTATGGATGCACAAGCTACAGCTTCACCTGAGGCTATGGCTGCTGCCGCTGATTCCGTCGATTTACAGCCGGGAATTTAATACGACTCACTATAGGGAGACCTCATCTTTGACATGAGCGATGACAAGAGCGTGGAGTCCTCGGtat                                                                                                                         >  1:392231/1‑299 (MQ=255)`  
                                                                                                                                                                                    `gATATTAACCTTGCGATGATTAAGTTACGTATTGCAAACGCTATCGGTATTGACACTTCTGGTATTCTACTCACCGAAGAACAGAAGCAACAGAAGATGGCCCAACAGTCTATGCAAATTGGGATGGATAATGGTGCTGCTGAGCTGGCTCAATGTATGGCTGCACAAGCTACAGCTTCACCTGAGGCTATGGATGCTGCCGCTGATTCCGTAGGTTTAAAGCAGGGAATTTAATACGAATCACTATAGGGAGACCTCATCTTTGAAATGAGCGATGACAAGAGTTTGGAGTCCTGGGttt                                                                                                                         >  1:400799/1‑299 (MQ=255)`  
                                                                                                                                                                                     `atatTAACCTTGCGATGATTAAGTTACGTATTGCCAACGCTATCGGTATTGACACTTCTGGTATTCTACTCACCTAATAACAGCATCAACAGAAGATGGCCCAACAGTCTATGCATTTTTGTATGGATACTTTTGCTGCTGCTCTTCCTCAAGGTATGTCTGCACAAGCTACTGCTTCACCTGAGCCTTTGGCTGCTGCCGCTGATTCCGTAGGTTTACAGCCTGGTATTTAATACGACTCACTATATGGATACCTCATCTTTGAAATTAGCGATGACAAGAGGTTGGTTTCCTCGTTCtt                                                                                                                        >  1:175354/1‑301 (MQ=255)`  
                                                                                                                                                                                      `tatTAACCTTGCGATGATTAAGTTACGTATTGCCAACGCTATCGGTATTGACACTTCTGTTCTTCTACTCACCGAAGAACAGAAGCAATAGAAGATGGCCCAACAGTCTATGCAACTTGGTATGGATCATGGTGCTGCTGCGCTGGCTCAAGGTATGGCTGCACAAGCTACAGCTTCACCTGAGGCTCTGGCTGCTGCCGCTGATTCCGTAGGTTTACAGCCTGGCCTTTAATACGACTCACTATAGGCACACCTCATCTTCTAAATGAGCGATGCCAAGCGGTTGGAGTCCTCGGTCTTc                                                                                                                       >  1:321783/1‑301 (MQ=255)`  
                                                                                                                                                                                         `tAACCTTGCGATGATTAAGTTACGTATTGCCAACGCTATCGGTATTGACACTTCTGGTATTCTACTCACCGAAGAACAGAAGCAACAGAAGATGGCCCAACAGTCTATGCAAATTGGTATGGATAATGGTGCTGCTGCGCTGGCTCAAGGTATGGCTGCACAAGCTACAGCTTCACCTGAGGCTATGGCTGCTGCCGCTGATTCCGTAGGTTTACAGCCGGGAATTTAATACGACTCACTATAGGGAGACCTCATCTTTGAAATGAGCGATGACAAGAGGTTGGAGTCCTCGGTCTTCCTg                                                                                                                    <  1:198282/301‑1 (MQ=255)`  
                                                                                                                                                                                         `tAACCTTGCGATGATTAAGTTACGTATTGCCAACGCTATCGGTATTGACACTTCTGGTATTCTACTCACCGAAGAACAGAAGCAACAGAAGATGGCCCAACAGTCTATGCAAATTGGTATGGATAATGGTGCTGCTGCGCTGGCTCAAGGTATGGCTGCACAAGCTACAGCTTCACCTGAGGCTATGGCTGCTGCCGCTGATTCCGTAGGTTTACAGCCGGGAATTTAATACGACTCACTATAGGGAGACCTCATCTTTGAAATGAGCGATGACAAGAGGTTGGAGTCCTCGGTCTTCCTg                                                                                                                    <  1:452381/301‑1 (MQ=255)`  
                                                                                                                                                                                                     `gatTAAGTTACGTATTGCCAACGCTATCGGTATTGACACTTCTGGTATTCTACTCACCGAAGAACAGAAGCAACAGAAGATGGCCCAACAGTCTATGCAAATTGGTATGGATAATGGTGCTGCTGCGCTGGCTCAAGGTATGGCTGCACAAGCTACAGCTTCACCTGAGGCTATTGCTGCTGCCGCTGATTCCGTAGGTTTACAGCCGGGAATTTAATACGACTCACTATAGGGAGACCTCATCTTTGAAATGAGCGATGACAAGAGGTTGGAGTCCTCGGTCTTCCTGTAGTTCAACttt                                                                                                        >  1:13006/1‑301 (MQ=255)`  
                                                                                                                                                                                                      `atTAAGTTACGTATTGCCAACGCTATCGGTATTGACACTTCTGGTATTCTACTCACCGAAGAACAGAAGCAACAGAAGATGGCCCAACAGTCTATGCAAATTGGTATGGATAATGGTGCTGCTGCGCTGGCTCAAGGTATGGCTGCACAAGCTACAGCTTCACCTGAGGCTATGGCTGCTGCCGCTGATTCCGTAGGTTTACAGCCGGGAATTTAATACGACTCACTATAGGGAGACCTCATCTTTGAAATGAGCGATGACAAGAGGTTGGAGTCCTCGGTCTTCCTgctgtctcttatac                                                                                                       >  1:457295/1‑288 (MQ=255)`  
                                                                                                                                                                                                         `atttgtataagagacagcGCTATCGGTATTGACACTTCTGGTATTCTACTCACCGAAGAACAGAAGCAACAGAAGATGGCCCAACAGTCTATGCAAATTGGTATGGATAATGGTGCTGCTGCGATGGCTCAAGGTATGGCTGCACAAGCTACAGCTTCACCTGAGGCTATGGCTGCTGCCGCTGATTCCGTAGGTTTACAGCCGGGAATTTAATACGACTCACTATAGGGAGACCTAATCTTTGAAATGAGCGATGACAAGAGGTTGGAGTCCTCGGTCTTCCGGTAGTTCAACTTTAAgg                                                                                                    <  1:371413/284‑1 (MQ=255)`  
                                                                                                                                                                                                         `aaGTTACGTATTGCCAACGCTATCGGTATTGACACTTCTGGTATTCTACTCACCGAAGAACAGAAGCAACAGAAGATGGCCCAACAGTCTATGCAAATTGGTATGGATAATGGTGCTGCTGCGCTGGCTCAAGGTATGGCTGCACAAGCTACAGCTTCACCTGAGGCTATGGCTGCTGCCGCTGATTCCGTAGGTTTACAGCCGGGAATTTAATACGACTCACTATAGGGAGACCTCATCTTTGAAATGAGCGATGACAAGAGGTTGGAGTCCTCGGTCTTCCTGTAGTTCAACTTTAAgg                                                                                                    >  1:412480/1‑301 (MQ=255)`  
                                                                                                                                                                                                          `aGTTACGTATTGCCAACGCTATCGGTATTGACACTTCTGGTATTCTACTCACCGAAGAACAGAAGCAACAGAAGATGGCCCAACAGTCTATGCAAATTGGTATGGATAATGGTGCTGCTGCGCTGGCTCAAGGTATGGCTGCACAAGCTACAGCTTCACCTAAGGCTATGGCTGCTGCCGCTGATTCCGTAGGTTTACAGCCGGGAATTTAATACGACTCACTATAGGGAGACCTCATCTTTGAAATGAGCGATGACAAGAGGTTGGAGTACTCGGTCTTCCTGTAGTTCAACTTTAAGga                                                                                                   >  1:136218/1‑301 (MQ=255)`  
                                                                                                                                                                                                           `gTTACGTATTGCCAACGCTATCGGTATTGACACTTCTGGTATTCTACTCACCGAAGAACAGAAGCAACAGAAGATGGCCCAACAGTCTATGCAAATTGGTATGGATAATGGTGCTGCTGCGCTGGCTCAAGGTATGGCTGCACAAGCTACAGCTTCACCTGAGGCTATGGCTGCTGCCGCTGATTCCGTAGGTTTACAGCCGGGAATTTAATACGACTCACTATAGGGAGACCTCATCTTTGAAATGAGCGATGACAAGAGGTTGGAGTCCTCGGTCTTCCTgctgtctcttatacacatc                                                                                                  >  1:375495/1‑283 (MQ=255)`  
                                                                                                                                                                                                                     `gacagcGCTATCGGTATTGACACTTCTGGTATTCTACTCACCGAAGAACAGAAGCAACAGAAGATGGCCCAACAGTCTATGCAAATTGGTATGGATAATGGTGCTGCTGCGCTGGCTCAAGGTATGGCTGCACAAGCTACAGCTTCACCTGAGGCTATGGCTGCTGCCGCTGATTCCGTAGGTTTACAGCCGGGAATTTAATACGACTCACTATAGGGAGACCTCATCTTTGAAATTAGCGATGACAAGAGGTTGGAGTCCTCGGTCTTCCTGTAGTTCAACTTTAAGGAGACaataataa                                                                                        <  1:104146/296‑1 (MQ=255)`  
                                                                                                                                                                                                                         `aCGCTATCGGTATTGACACTTCTGGTATTCTACTCACCGCAGAACAGAAACAACAGAAGATGGCCCAACAGTCTATGCAAATTGGTATGGATAATGGTGCTGCTGCGCTGGCTCAAGGTATGGCTGCACAAGCTACAGCTTCACCTGAGGCTATGGCTGCTGCCGCTGATTCCGTAGGTTTAAAGCCGGGAATTTAATACGACTCACTATAGGGAGACCTCATCTTTGAAATGAGCGATGACAAGAGGTTGGAGTCCTCGGTCTTCCTGTAGTTAAACTTTAAGGATACAATAATAATGGc                                                                                    <  1:202205/301‑1 (MQ=255)`  
                                                                                                                                                                                                                         `aCGCTATCGGTATTGACACTTCTGGTATTCTACTCACCGAAGAACAGAAGCAACAGAAGATGGCCCAACAGTCTATGCAAATTGGTATGGATAATGGTGCTGCTGCGCTGGCTCAAGGTATGGCTGCACAAGCTACAGCTTCACCTGAGGCTATGGCTGCTGCCGCTGATTCCGTAGGTTTACAGCCGGGAATTTAATACGACTCACTATAGGGAGACCTCATCTTTGAAATGAGCGATGACAAGAGGTTGGAGTCCTCGGTCTTCCTGTAGCTCAACTTTAAGGAGACAATAATAATGGc                                                                                    <  1:311940/301‑1 (MQ=255)`  
                                                                                                                                                                                                                          `cGCTATCGGTATTGACACTTCTGGTATTCTACTCACCGAAGAACAGAAGCAACAGAAGATGGCCAAACAGTCTATGCAAATTGGTATGGATAATGGTGCTGCTGCGCTGGCTCAAGGTATGGCTGCACAAGCTACAGCTTCACCTGAGGCTATGGCTGCTGCCGCTGATTCCGTAGGTTTACAGCCGGGAATTTAATACGACTCACTATAGGGACACCTCATCTTTGAAATGAGCGATGACAAGAGGTTGGAGTCCTCGGTCTTCCTGTAGTTCAACTTTAAGGAGACAATAATAATGGCt                                                                                   >  1:447916/1‑301 (MQ=255)`  
                                                                                                                                                                                                                              `aTCGGTATTGACACTTCTGGTATTCTACTCACCGAAGAACAGAAGCAACAGAAGATGGCCCAACAGTCTATGCAAATTGGTATGGATAATGGTGCTGCTGCGCTGGCTCAAGGTATGGCTGCACAAGCTACAGCTTCACCTGAGGCTATGGCTGCTGCCGCTGATTCCGTAGGTTTACAGCCGGGAATTTAATACGACTCACTATAGGGAGACCTCCTCTTTGCAATGAGCGATGACAAGAGGTTGGAGTCCTCGGTCTTCCTGTAGTTCAACTTTACGGAGACaataataactgtctctt                                                                               >  1:245350/1‑292 (MQ=255)`  
                                                                                                                                                                                                                                     `aagagacagcTGGTATTCTACTCACCGAAGAACAGAAGCAACAGAAGATGGCCCAACAGTCTATGCAAATTGGTATGGATAATGGTGCTGCTGCGCTGGCTCAAGGTATGGCTGCACAAGCTACAGCTTCACCTGAGGCTATGGCTGCTGCCGCTGATTCCGTAGGTTTACATCCGGGAATTTAATACGACTCACTATAGGGAGACCTCATCTTTGAAATGAGCGATGACAAGAGGTTGGAGTCCTCGGTCTTCCTGTAGTTCAACTTTAAGGAGACAATAATAATGGCTGAATCTAATGc                                                                        <  1:192347/292‑1 (MQ=255)`  
                                                                                                                                                                                                                                       `gACACTTCTGGTATACGACGCACCGAAGAACAGAAGCAACAGAAGATGGCCCAACAGTCTATGCAAATTGGTATGGATAATGGTGCTGCTGCGCTGGCTCAAGGTATGGCTGCACAAGCTACAGCTTCACCTGAGGCTATGGCTGCTGCCGCTGATTCCGTAGGTTTACAGCCGGGAATTTAATACGACTCACTATAGGGAGACCTCATCTTTGAAATGAGCGATGACAAGAGGTTGGAGTCCTCGGTCTTCCTGTAGTTCAACTTTAAGGAGACAATAATAATGGCTGAATCTAATGCAg                                                                      <  1:420412/301‑1 (MQ=255)`  
                                                                                                                                                                                                                                         `cacTTCTGGTATTCTACTAACCGAAGAACAGAAGCAACAGAAGATGGCCCAACAGTCTATGCAAATTGGTATGGATAATGGTGCTGCTGCGCTGGCTCAAGGTATGGCTGCACAAGCTACAGCTTCACCTGAGGCTATGGCTGCTGCCGCTGATTCCGTAGGTTTACAGCCGGGAATTTCATACGACTCACTATAGGGAGACCTCATCTTTTAAATGAGCGATGACAAGAGGTTGGAGTCCTCGGTCTTCCTGTAGTTCAACTTTAAGGAGACAATAATAATGGCTGAATCTAATGCAGAc                                                                    >  1:154567/1‑301 (MQ=255)`  
                                                                                                                                                                                                                                          `ttcggtgagttgaataccACCTAATAACGGAAGCAACAGAATGTGGCCCAACAGTCTATGCAAATTGGTATGGATAATGGTGCTGCTGCGCTGTCTCAAGGTATGGCTGCACCAGCTACAGCTTCCCCTGAGGCTATGGCTGCTTCCGCTGATTCCGTAGGTTTACAGCCGGGAATTTAATACGACTCACTATAGGGAGACCTCATCTTTGAAATGAGCGATGACAAGAGGTTGGAGTCCTCGGTCTTCCTGTAGTTCAACTTTAAGGAGACAATAATAATGGCTGAATCTAATGCAGACg                                                                   <  1:158885/284‑1 (MQ=255)`  
                                                                                                                                                                                                                                          `agacagggTATTCTACTCACCGAAGAACAGAAGCAACAGAAGATGGCCCAACAGTCTATGCAAATTGGTATGGATAATGGTGCTGCTGCGCTGGCTCAAGGTATGGCTGCACAAGCTACAGCTTCACCTGAGGCTATGGCTGCTGCCGCTGATTCCGTAGGTTTACAGCCGGGAATTTAATACGACTCACTATAGGGAGACCTCATCTTTGAAATGAGCGATGACAAGAGGTTGGAGTCCTCGGTCTTCCTGTAGTTCAACTTTAAGGAGACAATAATAATGGCTGAATCTAATGCAGACg                                                                   <  1:22292/295‑1 (MQ=255)`  
                                                                                                                                                                                                                                           `cTTCTGGTATTCTACTCACCGAAGAACAGAAGCAACAGAAGATGGCCCAACAGTCTATGCAAATTGGTATGGATAATGGTGCTGCTGCGCTGGCTCAAGGTATGGCTTCCCAAGCTACAGCTTCACCTGAGGCTATGGCTGCTGCCGCTGATTCCGTAGGTTTACAGCCGGGAATTTAATACGACTCACTATAGGGAGACCTCATCTTTGAAATGAGCGCTGACAAGAGGTTGGAGTCCTCGGTCTTCCTGTAGTTCAACTTTAAGTAGACAATAATAATGGCTGAATCTTATGCATAcat                                                                  >  1:59730/1‑299 (MQ=255)`  
                                                                                                                                                                                                                                           `cTTCTGGTATTCTACTCACCGAAGAACAGAAGCAACAGAAGATGGCCCAACAGTCTATGCAAATTGGTATGGATAATGGTGCTGCTGCGCTGGCTCAAGGTATGGCTGCACAAGCTACAGCTTCACCTGAGGCTATGACTGCTGCCGCTGATTCCGTAGGTTTACAGCCGGGAATTTAATACGACTCACTATAGGGAGACCTCATCTTTGAAATGAGCGATGACAAGAGGTTGGAGTCCTCGGTCTTCCTGTAGTTCAACTTTAAGGAGACAATAATAATGGCTGAATCTAATGCAGACGt                                                                  >  1:241182/1‑301 (MQ=255)`  
                                                                                                                                                                                                                                             `tCTGGTATTCTACTCACCGAAGAACAGAAGCAACAGAAGATGGCCCAACAGTCTATGCAAATTGGTATGGATAATGGTGCTGCTGCGCTGGCTCAAGGTATGGCTGCACAAGCTACAGCTTCACCTGAGGCTATGGCTGCTGCCGCTGATTCCGTAGGTTTACAGCCGGGAATTTAATACGACTCACTATAGGGAGACCTCATCTTTGAAATGAGCGATGACAAGAGGTTGGAGTCCTCGGTCTTCCTGTAGTTCAACTCTAAGGAGACAATAATAATGGCTGAATCTAATGcctgtctct                                                                >  1:444930/1‑293 (MQ=255)`  
                                                                                                                                                                                                                                                 `gTATTCTACTCACCGAAGAACAGAAGCAACAGAAGATGGCCCAACAGTCTATGCAAATTGGTATGGATAATGGTGCTGCTGCGCTGGCTCAAGGTATGGCTGCACAAGCTACAGCTTCACCTGAGGCTATGGCTGCTGCCGCTGATTCCGTAGGTTTACAGCCGGGAATTTAATACGACTCACTATAGGGAGACCTCATCTTTGAAATGAGCGATGACAAGAGGTTGGAGTCCTCGGTCTTCCTGTAGTTCAACTTTAAGGAGACAATAATAATGTCTGAATCTAATGCAGACGTATATga                                                            >  1:117906/1‑300 (MQ=255)`  
                                                                                                                                                                                                                                                 `gTATTCTACGCACCGAAGAACAGAAGCAACAGAAGATGGCCCAACAGTCTATGCAAATTGGTATGGATAATGGTGCTGCTGCGCTGGCTCAAGGTATGGCTGCACAAGCTACAGCTTCACCTGAGGCTATGGCTGCTGCCGCTGATTCCGTAGGTTTACAGCCGGGAATTTAATACGACTCACTATAGGGAGACCTCATCTTTGAAATGAGCGATGACAAGAGGTTGGAGTCCTCGGTCTTCCTGTAGTTCAACTTTAAGGAGACAATAATAATGGCTGAATCTAATGCAGACGTATATGc                                                            <  1:148837/301‑1 (MQ=255)`  
                                                                                                                                                                                                                                                 `aaagaaacagcACCGAAGAACAGAGGCAACAGAAGATGGCCCAACAGTCTATGCAAATTGGTATGGATAATGGTGCTGCTGCGCTGGCGCAAGGTATGGCTGCACAAGCTACAGCTTCACCTGAGGCTATGGCTGCTGCCGCTGATTCCGTAGGTTTACAGCCGGGAATTTAATACGACTAACTATAGGGAGACCTCATCTTTGAAATGAGCGATGACAAGAGGTTGGAGTCCTCGGTCTTCCTGTAGTTCAACTTTAAGGAGACAATAATAATGGCTGAATCTAATGCAGACGTATATGc                                                            <  1:316739/291‑1 (MQ=255)`  
                                                                                                                                                                                                                                                   `ttcctaataaCCGAAGAACAGAAGAAACAGAAGAGGGCCCAACAGTCTATGAAAATTGGTATGGATAATGGTGCGGCTGCGCTGGCTCAAGGTATGGCTGCACAAGCTACAGCTTCACCTTAGGCTATGGCTGCTGCCGCTGATTTCGTAGGTTTACAGCCGGGAATTTAATACGACTCACTATAGGGAGACCTCATCTTTGAAATGAGCGATGACAAGAGGTTGGAGTCCTCGGTCTTCCTGTAGTTCAACTTTAAGGAGACAATAATAATGGCTGAATCTAATGCAGACGTATATGCAt                                                          <  1:416605/292‑1 (MQ=255)`  
                                                                                                                                                                                                                                                    `tcctcatcacggactaacagaagaaacagaagaGTGAGAGAGAGTATATGAAAATTGGTAGGTATAATGGTGCTGCTGCGCTGGATGAAGGTATGGATGAACAAGCTACATCTTCAACTGAGGCTATGGCTGCTGCCGTTGATTCCCTATGTTTACAGCCGGGAATTTACTACGACTCACTATAGGGAGACCTCACCTTTGAAATTAGCGATGACAAGAGTTTGGAGTCCTCGGTCTTCCTGTAGTTCAACTTTAAGGAGACAATAATAATGGCTGAATCTAATGCAGACGTATATGCATc                                                         <  1:77794/286‑1 (MQ=255)`  
                                                                                                                                                                                                                                                        `aCTCACCGAAGAACAGAAGCAACAGAAGATGGCCCAACAGTCTATGCAAATTGGTATGGATAATGGTGCTGCTGCGCTGGCTCAAGGTATGGCTGCACAAGCTACAGCTTCACCTGAGGCTATGGCTGCTGCCGCTGATTCCGTAGGTTTACAGCCGGGAATTTAATACGACTCACTATAGGGAGACCTCATCTTTGAAATGAGCGATGACAAGAGGTTGGAGTCCTCGGTCTTCCTGTAGTTCAACTTTAAGGAGACAATAATAATGGCTGAATCTAATGCAGACGTATATGCATCtttt                                                     >  1:72559/1‑301 (MQ=255)`  
                                                                                                                                                                                                                                                        `aCTCACCGAAGAACAGAAGCAACAGAAGATGGCCCAACAGTCTATGCAAATTGGTATGGATAATGGTGCTGCTGCGCTGGCTCAAGGTATGGCTGCACAAGCTACAGCTTCACCTGAGGCTATGGCTGCTGACGCTGATTCCGTAGGTTTACAGCCGGGAATTTAATACGACTCACTATAGGGAGACCTCATCTTTGAAATGAGCGATGACAAGAGGTTGGAGTCCTCGGTCTTCCTGTAGTTCAACTTTAAGGAGACAATAATAATGGCTGAATCtgtctcttatacacatctccgagcc                                                     >  1:50123/1‑277 (MQ=255)`  
                                                                                                                                                                                                                                                            `aCCGAAGAACAGAAGCAACAGAAGATGGCCCAACAGTCTATGCAAATTGGTATGGATAATGGTTCTGCTGCGCTGGCTCAAGGTATGGTTGCACAAGCTACAGCTTCACCTGAGGCTATGGCTGCTGCCGCTGATTCCGTAGGTTTACAGCCGGGAATTTAATACGACTCACTATAGGGAGACCTCATCTTTGAAATGAGCGATGACAAGAGGTTGGAGTCCTCGGTCTTCCTGTAGTTCAACTTTAAGGAGACAATAATAATGGCTGAATCTAATGCAGACGTATATGCATCTTTTGGCg                                                 <  1:343944/301‑1 (MQ=255)`  
                                                                                                                                                                                                                                                             `ccGAAGAACAGAAGCAACAGAAGATGGCCCAACAGTCTATGCAAATTGGTATGGATAATGGTGCTGCTGCGCTGGCTCAAGGTATGGCTGCACAAGCTACAGCTTCACCTGAGGCTATGGCTGCTGCCGCTGATTCCGTAGGTTTACAGCCGGGAATTTAATACGACTCACTATAGGGAGACCTCATCTTTGAAATGAGCGATGACAAGAGGTTGGAGTCCTCGGTCTTCCTGTAGTTCAACTTTAAGGAGACAATAATAATGGCTGAATCTAATGCAGACGTATATGCATCTTTTGGCGt                                                >  1:334746/1‑301 (MQ=255)`  
                                                                                                                                                                                                                                                             `ccGAAGAACAGAAGCAACAGAAGATGGCCCAACAGTCTATGCAAATTGGTATGGATAATGGTGCTGCTGCGCTGGCTCAAGGTATGGCTGCACAAGCTACAGCTTCACCTGAGGCTATGGCTGCTGCCGCTGATTCCGTAGGTTTACAGCCGGGAATTTAATACGACTCACTATAGGGAGACCTCATCTTTGAAATGAGCGATGACAAGAGGTTGGAGTCCTCGGTCTTCCTGTAGTTCAACTTTAAGGAGACAATAATAATGGCTGAATCTAATGCAGACGTATATGCATCTTCTgtctc                                                >  1:425885/1‑297 (MQ=255)`  
                                                                                                                                                                                                                                                             `ccGAAGAACAGAAGCAACAGAAGATGGCCCAACAGTCTATGCAAATTGGTATGGATAATGGTGCTGCTGCGCTGGCTCAAGGTATGGCTGCACAAGCTACAGCTTCACCTGAGGCTATGGCTGCTGCCGCTGATTCCGTAGGTTTACAGCAGGGAATTTAATACGACTCACTATAGGGAGACCTCATCTTTGAAATGAGCGATGACAAGAGGTTGGAGTCCTCGGTCTTCCTGTAGTTCAACTTTAAGGAGACAATAATAATGGCTGAATCTAATGCAGACGTATATGCATCTTTTGGCGt                                                >  1:289932/1‑301 (MQ=255)`  
                                                                                                                                                                                                                                                             `ccGAAGAACAGAAGCAACAGAAGATGGCCCAACAGTCTATGCAAATTGGTATGGATAATGGTGCTGCTGCGCTGGCTCAAGGTATGGCTGCACAAGCTACAGCTTCACCTGAGGCTATGGCTGCTGCAGCTGATTCCGTAGGTTTACAGCCGGTAATTTAATACGACTCACTATAGGGAGACCTCATCTTTGAAATGAGCGATGACAAGAGGTTGGAGTCCTCGGTCTTCCTGTAGTTCAACTTTAAGGTGACAATAATAATGGCTGAATCTAATGCAGACAtatactgtctcttatacac                                                >  1:215904/1‑286 (MQ=255)`  
                                                                                                                                                                                                                                                                      `aGAAGCAACAGAAGATGGCCCAACAGTCTATGCAAATTGGTATGGATAATGGTGCTGCTGCGCTGGCTCAAGGTATGGCTGCACAAGCTACAGCTTCACCTGAGGCTATGGATGCTGCCGCTGATTCCGTAGGTTTACAGCCGGGAATTTAATACGACTCACTATAGGGAGACCTCATCTTTGAAATGAGCGATGACAAGAGGTTGGAGTCCTCGGTCTTCCTGTAGTTCAACTTTAAGGAGACAATAATAATGGCTGAATCTAATGCAGACGTATATGCATCTTTTGGCGTGAACTCCGc                                       <  1:26908/301‑1 (MQ=255)`  
                                                                                                                                                                                                                                                                           `cAACAGAAGATGGCCCAACAGTCTATGCAAATTGGTATGGATAATGGGGCTGCTGCGCTGGCTCAAGGTATGGCTGCACAAGCTACAGCTTCACCTGAGGCTATGGCTGCTGCCGCTGATTCCGTAGGTTTACAGCCGGGAATTTAATACGACTCACTATAGGGAGACCTCATCTTTGAAATGAGCGATGACAAGAGGTTGGAGTCCTCGGTCTTCCTGTAGTTCAACTTTAAGGAGACAATAATAATGGCTGAATCTAATGCAGACGTATATGCATCTTTTGGCGTGAACTCCGCTGTGa                                  <  1:121466/301‑1 (MQ=255)`  
                                                                                                                                                                                                                                                                           `aaaCAGAAGATGGCACAACAGACTATGCAAATTGGTATGGGTAATGGTGCTGCTGCGCTGGCTCAAGGTATGGCTGCACAAGCTACAGCCTCACCTGAGGCTATGGCTGCTGCCGCTGATTCCGTAGGTTTACAGCCGGGAATTTAATACGACTCACTATAGGGAGACCTCATCTTTGAAATGAGCGATGACAAGAGGTTGGAGTCCTCGGTCTTCCTGTAGTTCAACTTTAAGGAGACAATAATAATGGCTGAATCTAATGCAGACGTATATGCATCTTTTGGCGTGAACTCCGCTGTGa                                  <  1:260473/300‑1 (MQ=255)`  
                                                                                                                                                                                                                                                                             `aCAGAAGATGGCCCAACAGTCTATGCAAATTGGTATGGATAATGGTGCTGCTGCGCTGGCTCAAGGTATGGCTGCACAAGCTACAGCTTCACATGAGGCTATGGCTGCTGCCGCTGATTCCGTAGGTTAACAGCCGGGAATTTAATACGACTCACTATAGGGAGACCTCATCTTTGAAATGAGCGATGACAAGAGGTTGGAGTCCTCGGTCTTCCTGTAGTTCAACTTTAAGGAGACAATAATAATGGCTGAATCTAATGCAGACGTATATGCATCTTTTGGCGTGAACTCCGCTGTGATg                                >  1:96080/1‑301 (MQ=255)`  
                                                                                                                                                                                                                                                                                             `aaGTCTAGTCAAATTGGTATGGATAAGTGTGCTGCTGCGCTGGCTCAAGGTATGGCTGAACAAGCTAAAGCTTCACCTGAGGCTATGGGTGCTGCCGCTGATTCCGTAGGTTTACAGCTGGGAATTTAATACGACTCACTATAGGGATACCTCATCTTTGAAATGAGCGATGACAAGAGGTTGGAGTCCGCGGTCTTCCTGTAGTTCAACTTTAAGGAGACAATAATAATGGCTGAATCTAATGCAGACGTATATGCATCTTTTGGCGTGAACTCCGCTGTGATGTCTGGTGGTTCCGTTg                <  1:39572/300‑1 (MQ=255)`  
                                                                                                                                                                                                                                                                                                   `aTGCAAATTGGTATGGATAATGGTGCTGCTGCGCTGGCTCAAGGTATGGCTGCACAAGCTACAGCTTCACCTGAGGCTATGGCTGCTGCCGCTGATTCCGTAGGTTTACAGCCGGGAATTTAATACGACTCACTATAGGGAGACCTCATCTTTGAAATGAGCGATGACAAGAGGTTGGAGTCCTCGGTCTTCCTGTAGTTCAACTTTAAGGAGACAATAATAATGGCTGAATCTAATGCAGACGTATATGCATCTTTTGGCGTGAACTCCGCTGTGATGTCTGGTGGTTCCGTTGATGAAc          >  1:469903/1‑301 (MQ=255)`  

                                                                                                                                                                                                                                                                                                           `|`                                                                                                                                                                                                                                                                                                              
`CTGGTACGAGTGCTCTTGAAGCAACTACAAGCCACGCAACAGATTCCTGAGTTACCTAAGGAAGCCGTAGAGCCAACCATTAGTACAGGTCTGGAAGCAATTGGTCGAGGACAAGACCTTGATAAGCTGGAGCGGTGTGTCACTGCGTGGGCTGCACTGGCACCTATGCGGGACGACCCTGATATTAACCTTGCGATGATTAAGTTACGTATTGCCAACGCTATCGGTATTGACACTTCTGGTATTCTACTCACCGAAGAACAGAAGCAACAGAAGATGGCCCAACAGTCTATGCAAATGGGTATGGATAATGGTGCTGCTGCGCTGGCTCAAGGTATGGCTGCACAAGCTACAGCTTCACCTGAGGCTATGGCTGCTGCCGCTGATTCCGTAGGTTTACAGCCGGGAATTTAATACGACTCACTATAGGGAGACCTCATCTTTGAAATGAGCGATGACAAGAGGTTGGAGTCCTCGGTCTTCCTGTAGTTCAACTTTAAGGAGACAATAATAATGGCTGAATCTAATGCAGACGTATATGCATCTTTTGGCGTGAACTCCGCTGTGATGTCTGGTGGTTCCGTTGAGGAACATGAGCAG  >  NC_001604.1/21437‑22036`

  

Alignment Legend

Aligned base mismatch/match (shaded by quality score): `ATCG/ATCG` `< 3 ≤` `ATCG/ATCG` `< 10 ≤` `ATCG/ATCG` `< 20 ≤` `ATCG/ATCG` `< 34 ≤` `ATCG/ATCG` `< 38 ≤` `ATCG/ATCG`

Unaligned base: `atcg`    Masked matching base: `atcg`    Alignment gap: `‑`    Deleted base: `‑`
